# Modulation of IRF7-driven transcription as a strategy to control HIV-1 latency

**DOI:** 10.3389/fimmu.2026.1735192

**Published:** 2026-03-16

**Authors:** Ifeanyi Jude Ezeonwumelu, Edurne Garcia-Vidal, Eudald Felip, Sara Cabrero-de las Heras, Bonaventura Clotet, Roger Badia, Ester Ballana, Eva Riveira-Muñoz, Maria Nevot

**Affiliations:** 1 IrsiCaixa, Badalona, Spain; 2 Health Research Institute Germans Trias i Pujol (IGTP), Hospital Universitari Germans Trias i Pujol, Universitat Autònoma de Barcelona, Badalona, Spain; 3 Institut Català d’Oncologia, Hospital Universitari Germans Trias i Pujol, Badalona, Spain; 4 Centro de Investigación Biomédica en Red de Enfermedades Infecciosas, CIBERINFEC, Madrid, Spain

**Keywords:** block and lock strategy, HIV-1 latency, IRF7 transcription factor, Janus kinase 2 inhibitor (JAK2i), latency-promoting agents (LPA)

## Abstract

**Background:**

The persistence of latent HIV-1 reservoirs remains a major barrier to achieving a cure for HIV. While latency-reversing agents (LRAs) have been extensively studied, latency-promoting agents (LPAs) offer a complementary strategy to silence viral transcription and prevent immune activation. Here, we propose that modulation of IRF7-driven transcription may represent a novel approach to control HIV-1 latency, by characterizing the role of the Janus kinase 2 inhibitor (JAK2i) pacritinib as a novel latency-promoting agent (LPA).

**Methods:**

The impact of JAK2i on HIV-1 reactivation, immune activation, and IRF7 expression were evaluated in lymphoid and myeloid HIV-1 latency models, as well as *ex vivo* CD4^+^ T cells from ART-suppressed individuals. IRF7 modulation was assessed by qRT-PCR and immunoblotting, and its functional role confirmed through LTR transactivation assays and IRF7 overexpression. Co-immunoprecipitation was used to detect IRF7–Tat interaction. Whole transcriptomic profiling with pathway analysis were performed to identify the molecular signatures associated with JAK2i treatment.

**Results:**

Pacritinib effectively suppressed HIV-1 latency reversal induced by LRAs without triggering immune activation. Mechanistically, pacritinib downregulated IRF7 expression at both transcript and protein levels, correlating with reduced HIV-1 transcription. Overexpression of IRF7 restored LTR transactivation, confirming its central role in HIV-1 transcription and latency. Co-immunoprecipitation assays revealed a direct interaction between IRF7 and the viral transactivator Tat. Furthermore, pacritinib selectively inhibited multiply spliced HIV-1 transcripts, suggesting a blockade at late transcriptional stages.

**Conclusion:**

Pacritinib acts as a potent LPA by silencing HIV-1 transcription through IRF7 downregulation, supporting a promising “block and lock” strategy for functional cure approaches. Targeting IRF7 may enable durable suppression of the viral reservoir without immune activation, supporting the development of “block and lock” therapies.

## Introduction

Despite extensive resources and dedicated efforts, human immunodeficiency virus type-1 (HIV-1) infection remains an incurable disease to date (1, 2). The introduction of antiretroviral therapy (ART) represented a significant breakthrough, transforming HIV-1 infection into a chronic condition, particularly in regions where favorable social, geographical, and economic factors facilitate access and adherence to treatment for people living with HIV (PWH) (3). ART effectively suppresses HIV-1 replication to undetectable levels; however, it does not achieve complete viral eradication due to the persistence of long-lived reservoirs in which the HIV genome remains stably integrated in a transcriptionally silent state (4). These reservoirs can reactivate by multiple factors, including ART discontinuation and clonal activation causing viral rebound (5) or low-level often undetectable residual replication (6, 7). Therefore, life-long adherence to ART is required to sustain viral suppression for most of PWH. Nevertheless, a small subset of individuals can sustain long-term viral control of the infection maintaining viremia undetectable in the absence of ART, such as elite controllers and long-term non-progressors (8, 9). Different strategies have been attempted to either completely eradicate or minimize the size of the HIV reservoir and gain control of viral replication after ART discontinuation, thus paving the way for a true functional cure (10). The “shock and kill” strategy aims to induce the transcription of the silent genome upon treatment with latency reversing agents (LRAs) to produce viral particles that expose HIV-1 infected cells to immune clearance (11, 12). Mechanistically inverse, the “block and lock” strategy pursues HIV-1 transcriptional silencing by using latency promoting agents (LPAs) to drive the provirus into a profoundly latent state, thereby preventing viral reactivation and sustained replication, even in the absence of ART (13). However, despite the promising *in vitro* results, none of the strategies has succeeded in an *in vivo* setting. Even under effective ART, PWH continue to experience persistent immune activation, immune exhaustion and accelerated aging, primarily due to ongoing production of HIV-1 antigens (14–16). Thus, therapeutic strategies capable of inhibiting HIV-1 transcription are crucial to prevent HIV-induced immune dysfunction and persistent activation until a safe, effective and broadly applicable strategy for eradicating HIV is developed.

Janus kinase inhibitors (JAKi) are pharmacological agents designed to selectively block intracellular signaling pathways initiated by IFN, cytokines and/or growth factors, which regulate immune responses and inflammatory processes (17). Initially approved for the treatment of rheumatoid arthritis and myelofibrosis, several JAKi, baricitinib, tofacitinib, filgotinib and ruxolitinib, are now being repurposed as immunomodulators. The immunomodulatory potential of JAKi could also be exploited in cure-based regimens for PWH thanks to their ability to inhibit HIV expression *in vitro* and *in vivo* (18) and to impede HIV production after latency reversal, while decreasing biomarkers associated with T-cell activation, immune dysregulation, and inflammation (19, 20). Recently, we have described a specific subclass of selective JAK2i which reverses HIV latency *in vitro* and *ex vivo* through an interferon-independent activation of interferon regulatory factor 7 (IRF7) mechanism (21). Moreover, we have shown that induction of IRF7 expression positively correlates with the latency reversal capacity of JAK2i. Here, we demonstrate the key role of IRF7 in HIV-1 transcription and latency, identifying also novel JAK2i that might be used as potent LPA, overall providing new strategies towards HIV eradication.

## Materials and methods

### Primary cultures and cell lines

The human cell lines HL60, Jurkat, MOLT-4 and U937 were obtained from ATCC (Gaithersburg, MD). HEK293T, J-Lat 8.4 and TZM-bl cell lines were obtained from the AIDS Reagent Program, National Institutes of Health (Germantown, MD). HL60, Jurkat, MOLT-4, U937 and J-Lat 8.4 cells were cultured in complete RPMI culture medium (RPMI 1640 medium supplemented with 10% FBS, 100 U/ml penicillin and 100 μg/ml streptomycin). HEK293T and TZM-bl cells were cultured in Dulbecco’s modified Eagle’s medium (DMEM), supplemented with 10% FBS, 100 U/ml penicillin and 100 μg/ml streptomycin. All cell cultures were maintained at 37 °C in a 5% CO2 incubator.

Buffy coats from healthy donors were obtained from the Catalan Blood and Tissue Bank. The samples were provided fully anonymized and untraceable, with the only information available being whether or not they had been screened for diseases. All donors gave informed consent during blood collection, and the procedures followed were in line with relevant guidelines, regulations, and the ethical principles outlined in the Declaration of Helsinki. PBMCs were obtained using a Ficoll-Paque density gradient centrifugation and were kept in complete RPMI 1640 medium supplemented with 10% heat-inactivated fetal FBS, 100 U/mL penicillin, 100 µg/mL streptomycin and rIL-2 (6.5 IU/mL), as described previously (22).

### Patients and samples

This study sampled a cohort of chronically infected PWH attending Hospital Germans Trias i Pujol, Badalona, Spain. Study participants were included if the individuals were older than 18 years old and had been on suppressive ART with undetectable plasma HIV-1 RNA levels (<40 copies/ml) for a minimum of two years. Six participants were male and one was female. The median age of the participants was 46 years (range 38-63). Frozen PBMCs from enrolled participants were isolated as described above and cryopreserved. Immunological and virological characteristics of all participants are found in **Supplementary Table 1**. All participants in the study provided informed consent, and the work was approved by the Scientific Committee of IrsiCaixa and the Ethics Committee of Hospital Germans Trias i Pujol (Ref. CEI PI-18–021). Thawed PBMCs were used to purify CD4+ T lymphocytes by negative selection (StemCell Technologies) and the purity was confirmed by flow cytometry.

### Viral strains

The envelope-deficient HIV-1 NL4–3 clone (HIG) encoding internal ribosome entry site (IRES)-green fluorescent protein (GFP) (NL4-3-GFP) [50] was pseudotyped with vesicular stomatitis virus G protein (VSV-G) by cotransfection of HEK293T cells using polyethylenimine (Polysciences) as previously described (23).

Three days after transfection, supernatants were harvested, filtered and stored at -80 °C. Viral stocks were concentrated using Lenti-X concentrator (Clontech). Viruses were titrated by infection of TZM-bl cells followed by GFP quantification by flow cytometry.

### Generation of non-clonal HIV latently infected cells and viral reactivation assays

Latently infected cells (J-HIG, HL-HIG, U-HIG and MOLT-HIG) were generated by infecting lymphoid (Jurkat and MOLT-4) and myeloid (HL60 and U937) cells with HIG virus and maintained in culture for 10 days to allow for the attrition of productively infected cells as previously described (21). HIV-1 reactivation was measured as the percentage of GFP + cells by flow cytometry 20 h post-incubation with the compounds, relative to untreated control (DMSO). For evaluation of cell death, cells were stained for 30 min with LIVE/DEAD™ Fixable Near-IR Dead Cell Stain Kit (Invitrogen) in PBS according to the manufacturer’s instructions. Flow cytometry assays were performed in a FACS LSR II or a FACSCanto II flow cytometer (BD Biosciences). The data was analyzed using the FlowJo software (BD Biosciences).

### Compounds and drug treatments

Antiretroviral drugs, 3-azido-3-deoxythymidine (zidovudine; AZT), used at 3 or 10 μM, raltegravir at 1 or 5 μM and efavirenz at 0.32 μM were obtained from the NIH AIDS Research and Reference Reagent Program. PMA (Sigma-Aldrich) was used at 50–100 ng/ml and ionomycin (Sigma-Aldrich) was used at a concentration of 1 µg/ml. Pacritinib was used at concentrations ranging between of 0.2-3 µM, fedratinib and vorinostat were used at 5 µM and were all purchased from Selleckchem. When used in combination with an LRA, pacritinib was added 2 hours prior to LRA exposure.

### *Ex vivo* reactivation of primary CD4+ T cells from PWH

Purified CD4+ T lymphocytes from HIV-1+ participants were kept in complete RPMI culture medium and preincubated with the pan-caspase inhibitor Q-VD-Oph (10 µM, Sigma-Aldrich) for 2 h. To evaluate the latency reactivation capacity, 0.5 milion CD4+ T lymphocytes were cultured with PMA + ionomycin with or without pacritinib at the indicated concentrations (1 µM pacritinib, and 50 ng/ml PMA + 1 µg/ml ionomycin). Cells were cultured in the presence of antiretrovirals (efavirenz, zidovudine and raltegravir) and maintained in 10 µM Q-VD-Oph for 72 h.

Upon treatment, freshly collected cell-culture supernatants were centrifuged for 1 hour at 25–000 *g* to pellet HIV particles. HIV-1 reactivation was determined by quantification of viral RNA in the supernatant as previously described (24). Briefly, viral RNAs were extracted using Viral RNA/DNA Mini kit (Invitrogen) and quantified using a nested real-time reverse transcription-polymerase chain reaction (RT-PCR). A Superscript III One-Step RT-PCR system (Invitrogen) was used to generate and pre-amplify viral RNA with the following primers: ULF1 (forward) 5’- ATG CCA CGT AAG CGA AAC TCT GGG TCT CTC TDG TTA GAC - 3’; UR1 (reverse) 5’- CCA TCT CTC TCC TTC TAG C -3’. The following cycling conditions were used: reverse transcription at 50°C for 30 min, denaturation at 94°C for 2 min, 16 cycles of amplification (94°C 15 s, 55°C 30 s, 68°C 1 min) and final elongation at 68°C for 5 min. Preamplified products were subjected to nested real-time PCR with the following primers and probe: LambdaT (forward) 5’- ATG CCA CGT AAG CGA AAC T - 3’; UR2 (reverse) 5’ - CTG AGG GAT CTC TAG TTA CC - 3’; UHIV Taqman 5’- 56-FAM/CAC TCA AGG/ZEN/CAA GCT TTA TTG AGG C/3IbkFQ/- 3’ on a QuantStudio 5 PCR system (Applied Biosystems). Serial dilutions of the HIV-1 NL43 strain were run in parallel with each experiment for the quantification of viral RNA.

### Immunophenotypic characterization of PBMCs by flow cytometry

Purified CD4+ T lymphocytes from PWH were stained with CD4+T cell phenotype defining markers for flow cytometry 72 h post-treatment with compounds: CD4-BV785, (Biolegend) and CD3-FITC (BD Biosciences). Immune activation was determined using CD69-BV650, CD25-APC (Biolegend) and HLA-DR-PeCy7 (BD Biosciences). Immunophenotyping of CD4+ T cell population was performed based on the gating strategy defined in **Supplementary Figure 1**: immune activation markers HLA-DR+, CD25+ and CD69+ CD4+ lymphocytes were gated on the live singlet CD3+CD4+ lymphocytes. Cells were washed and fixed in 1% formaldehyde before the analysis. Flow cytometry assays were performed in a FACS LSR II or a FACSCanto II flow cytometer (BD Biosciences). The data was analyzed using the FlowJo software (BD Biosciences).

### Quantitative RT-polymerase chain reaction

Total RNA was extracted using NucleoSpin RNA II kit (Macherey-Nagel) or the Maxwell^®^ HT simplyRNA Kit (Promega) and Total DNA was extracted using Magmax DNA multi-sample ultra 2.0 kit (appliedbiosystems) on a KingFisher™ Flex Purification System (Thermofisher Scientific) as recommended by the manufacturer. Reverse transcription was performed using the PrimeScript™ RT-PCR Kit (Takara) following manufacturer instructions. mRNA levels of all genes were measured by two-step quantitative RT-PCR and normalized to GAPDH mRNA expression using the DDCt method.

Viral RNA and integrated DNA quantification were performed as described before (25–28). For viral RNA quantification, the following primers and probe amplifying spliced *tat/rev/nef* mRNA were used: forward 5′-GGATCTGTCTCTGTCTCTCTCTCCACC-3′, reverse 5′-ACAGTCAGACTCATCAAGTTTCTCTATCAAAGCA-3′ and the dual-labeled fluorescent probe FAM 5′-TTCCTTCGGGCCTGTCGGGTCCC-3′ TAMRA. For integrated viral DNA quantification an Alu-Gag (HIV group-specific antigen) preamplification was performed by using the following primers: forward 5′-GCCTCCCAAAGTGCTGGGATTACAG-3′ and reverse 5′AGGGTTCCTTTGGTCCTTGT-3′; followed by a Gag amplification by using the following primers and probe: forward 5′-CAAGCAGCCATGCAAATGTT-3′, reverse 5′-TGCACTGGATGCAATCTATCC-3′, and probe FAM 5′-AAAGAGACCATCAATGAGGAAGCTGCAGA-3′ TAMRA. Primers and DNA probes were purchased from IDT (Coralville, IA) TaqMan Gene expression assays were from Thermofisher Scientific (Cat#433182: Hs00266705_g1-GAPDH, Hs01014809_g1-IRF7).

### Immunoblot

Treated cells were rinsed, lysed, subjected to SDS-PAGE and transferred to a polyvinylidene difluoride (PVDF) membrane as previously described (29). The following antibodies were used for immunoblotting: antirabbit and anti-mouse horseradish peroxidase-conjugated secondary antibodies (1:5000; Pierce); anti-GAPDH (1:2500; ab9485; Abcam); anti-pJAK2 (1:1000; 3774), anti-phosphoSTAT1 (1:1000; 9167), anti-IRF7 (1:1000; 4920), all from Cell Signaling. Blots were immersed in chemiluminescent substrate (SuperSignal West Pico Plus or Femto, Thermo Fisher Scientific), and signal was visualized using ChemiDoc MP imaging system (BIORAD). Full length Western blot images are shown in **Supplementary Figures 4A–D**).

### Transactivation assay of HIV-1 LTR promoter

HeLa TZM-bl cells harboring an integrated copy of HIV-1 LTR, controlling luciferase reporter gene expression was used for a viral transactivation assay. Cells were transfected with 2ugr of pUNO1_IRF7-HA or pUNO1-HA using Lipofectamine 3000 reagent (Invitrogen) in a 6-well plate following manufacturer instructions, and overexpression of IRF7 was confirmed by WB. 24h post-transfection, treatment with vorinostat, fedratinib or pacritinib was performed in the same well. 24h post-treatment, cells were counted and re-seeded in a 96-well white plate, and transactivation was then measured by a luciferase-based assay.

### Co-immunoprecipitation and Western blotting

HEK293T cells were harvested, washed in cold PBS, and lysed on ice with lysis buffer as described (30). After centrifugation at 14,000 rpm for 10 min at 4 °C, lysates were collected, and aliquots were resuspended in loading buffer, then subjected to SDS-PAGE and transferred to a PVDF membrane. The following antibodies were used for immunoblotting: horseradish peroxidase-conjugated secondary antibody (Pierce), anti-IRF7 anti- HA (Cell Signaling) and anti-FLAG-Tat (Invitrogen). Lysates from HEK293T cells were incubated with anti-FLAG antibodies covalently attached to agarose (anti-FLAG M2 Affinity Gel, Sigma) or anti HA- agarose antibodies (mouse monoclonal anti HA agarose Ab, Sigma) overnight at 4 °C on a rocking platform for FLAG-Tat fusion protein or HA-IRF7 fusion protein immunoprecipitation. Beads were collected by centrifugation at 3000 rpm for 5 min at 4 °C, extensively washed in lysis buffer, and resuspended in SDS loading buffer. The proteins were separated on a 4-20% SDS-polyacrylamide gel, transferred to a PVDF membrane, and analyzed by immunoblotting with the corresponding antibodies. Full length Western blot images are shown in **Supplementary Figure 4E**).

### RNA-sequencing and library preparation

The library was prepared as previously described (21). Briefly, RNA was extracted from previously treated (PMA, fedratinib or pacritinib) and untreated HL-HIG cells in triplicate. Samples were sent to Macrogen (Seoul, Republic of Korea) and, after determination of RNA integrity and passing the quality control checks, the RNA library was constructed using Illumina TruSeq Stranded mRNA LT Sample Prep Kit. Sequencing was performed using NovaSeq 6000 System with 150 bp paired-ends reads.

### Transcriptomic analysis

Transcriptomic analysis was performed as described previously (21) for the detection of differentially expressed genes (DEGs) and pathways between the untreated and treated conditions from the RNA-seq data. Briefly, aligned reads with low expression (at least one zero count) were filtered out. Afterwards, the reads were normalized using R Statistical Software (v4.4.0; R Core Team 2024) with the Relative Log Expression (RLE) method, as implemented in DESeq2 R library. Differential gene expression was expressed as Log2 Fold-change of the treated conditions compared to the untreated control using DESeq2 Wald test (31).

IRF7 response to RNA viruses networks were generated with QIAGEN IPA (QIAGEN Inc., https://digitalinsights.qiagen.com/IPA) (31) from the Canonical Pathway network “Activation of IRF by Cytosolic Pattern Recognition Receptors”. Gene and pathway expressions and predictions were generated by overlaying the DEG datasets from the previous transcriptomic analysis performed with R.

### Quantitative assessment of drug-drug interactions

To evaluate the interaction between PMA, ionomycin and pacritinib, we compared the observed effect of the drug combination with the predicted effect calculated using the Bliss Independence model, as previously described (32). Briefly, the predicted combined effect was calculated using the following equation: f_axy, P_ = f_ax_ + f_ay_ - (f_ax_ * f_ay_), where f_ax_ and f_ay_ correspond to the experimental effects of each individual drug alone.

The Bliss Independence index score (Δf_axy_) was then calculated by subtracting the predicted effect from the observed experimental effect of the combination, as follows: Δf_axy =_ f_axy, O_ - f_axy, P_.

A Δf_axy_ > 0 indicated that the observed effect is greater than that predicted by the model, suggesting a synergistic interaction between the compounds. Conversely, a Δf_axy_ < 0 indicates a lower-than-expected effect, implying antagonism. When Δf_axy_ = 0, the observed combined effect is consistent with that predicted by the Bliss Independence model, indicating no interaction between the compounds.

### Statistical analysis

Statistical significance for *in vitro* and *ex vivo* experiments was calculated using appropriate t-test in GraphPad Prism (v9.3.0). All experiments were performed in at least three independent replicates, and n values are provided in the figure legends. Plots were drawn using GraphPad Prism and R software.

## Results

### JAK2i pacritinib is a potent latency promoting agent.

**Figure 1:**
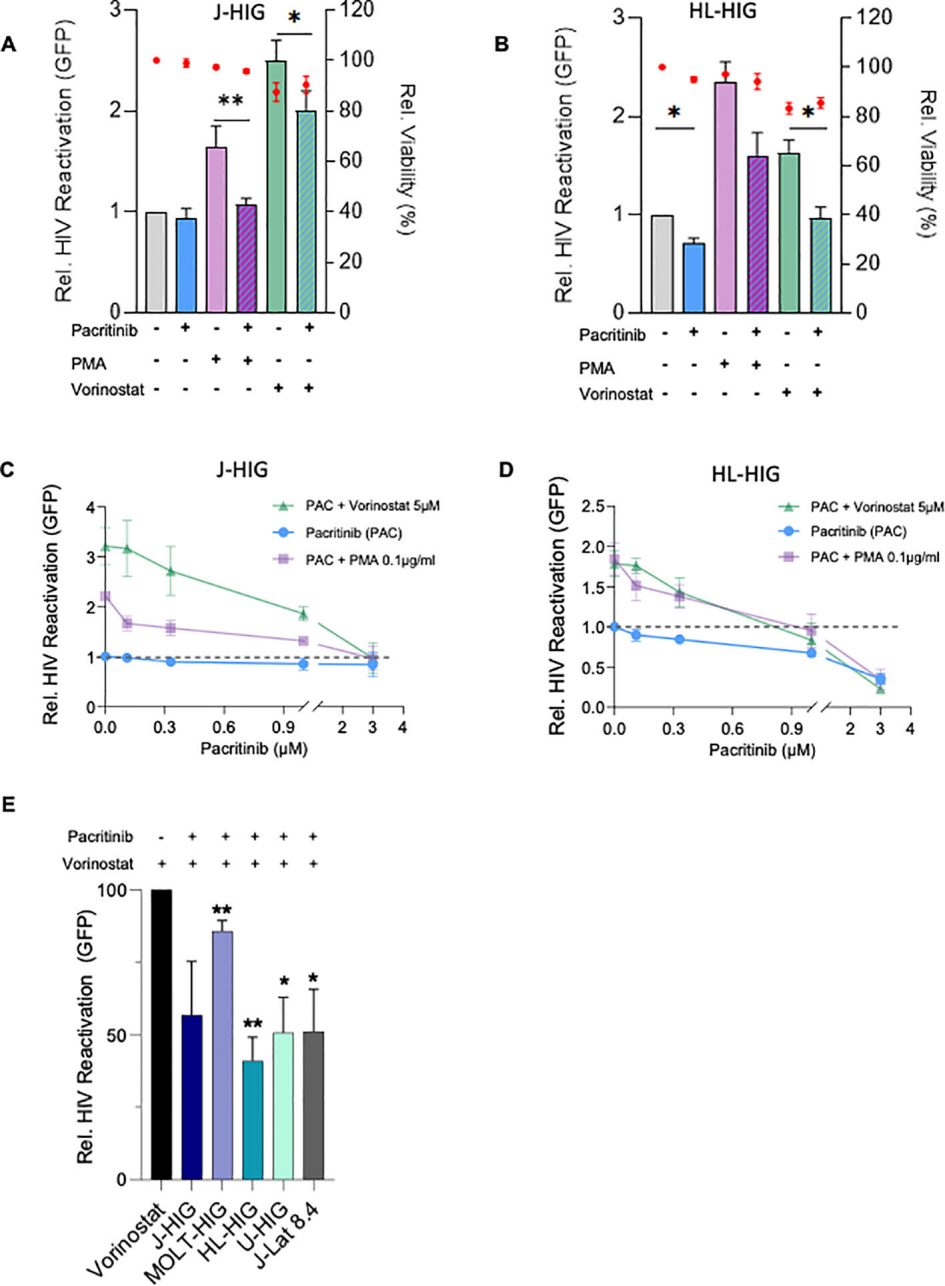
Pacritinib significantly blocked HIV-1 reactivation both, alone or in combination with LRAs, in *in vitro* models of HIV-1 latency. **(A, B)** Bar plots represent relative viral reactivation measured as the proportion of GFP+ cells 24h post incubation with pacritinib (1µM) alone or in combination with PMA (0.1µg/ml) and vorinostat (5µM) in J-HIG and HL-HIG respectively. Red dots represent relative cell viability of treatment conditions. Means ± SD values normalized to the untreated condition. **(C, D)** Expanded dose-response testing of pacritinib alone or in combination with PMA and vorinostat in J-HIG and HL-HIG respectively. Means ± SD values normalized to the untreated condition (dashed line). **(E)** Viral reactivation measured as in A with pacritinib (1µM) and vorinostat (5µM) with the additional HIV-1 latency models MOLT-HIG, U-HIG and J-Lat 8.4. Means ± SD values normalized to the vorinostat only condition in each model. Data obtained from at least three independent experiments *p<0.05; **p<0.005.

Building on previous data on latency modulating properties of JAK2i (21), the capacity of the JAK2 inhibitor pacritinib as a latency promoting agent (LPA) was evaluated. Our lymphoid J-HIG latency model was stimulated with PMA or vorinostat and reactivation of latent HIV-1 proviruses normalized to the untreated condition was determined by measuring GFP expression. Preincubation with a single dose of pacritinib (1 µM) treatment effectively blocked provirus reactivation induced by PMA (p=0.002) as well as by vorinostat (p=0.013), measured by a reduction of GFP+ cell population in the absence of cytotoxicity. No significant effect was observed in pacritinib treated unstimulated cells (**Figure 1A**). We further determined the inhibitory effect of pacritinib in the myeloid non-clonal latency model since, in addition to CD4+ T cells, cells from the monocyte/macrophage lineage may be another source of HIV-1 latent reservoirs (33, 34). As in J-HIG, pacritinib treatment significantly blocked HIV-1 latency reversal induced by vorinostat in the HL-HIG model (p=0.019) and, following a similar trend upon PMA induction. Interestingly, pacritinib also showed an inhibitory effect on spontaneous HIV-1 latency reversal in myeloid cells (p=0.003) (**Figure 1B**). Additionally, expanded dose-response testing of pacritinib, both alone and in combination with PMA and vorinostat, demonstrated a clear and dose-dependent inhibition of HIV-1 reversal induced by these LRAs in both J-HIG and HL-HIG models (**Figures 1C, D**). To further evaluate the activity of pacritinib as an LPA, we used it in combination with vorinostat in other non-clonal lymphoid (MOLT-HIG) and myeloid (U-HIG) models and in a lymphoid clonal model (J-Lat clone 8.4). In all of them, pacritinib significatively blocked vorinostat-induced latency reversal (**Figure 1E**).

**Figure 2:**
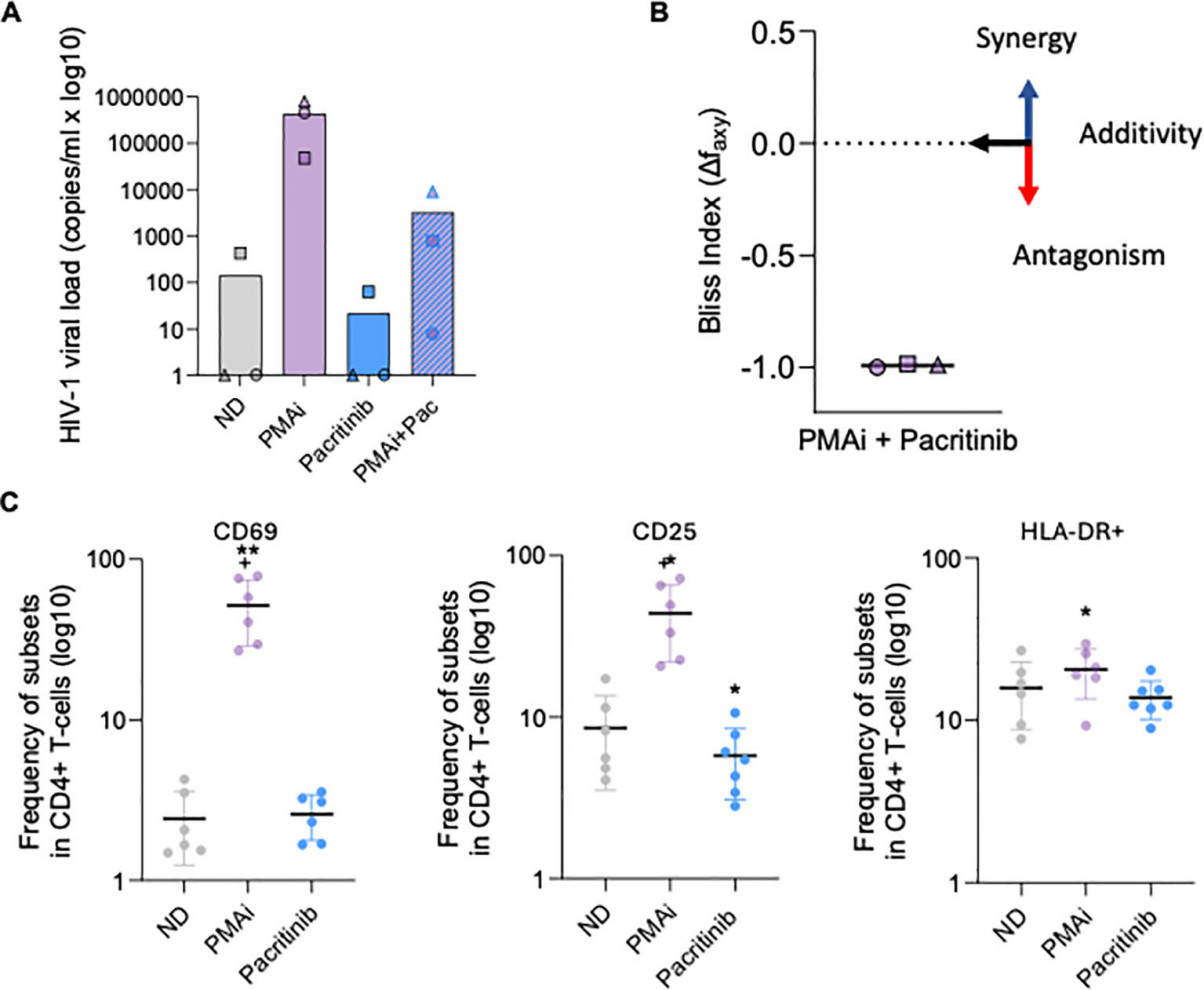
Pacritinib blocks HIV-1 reactivation both, alone or in combination with LRAs in *ex vivo* models of HIV-1 latency without immune activation induction. **(A)** Quantification of HIV-1 RNA in cell culture supernatant of isolated CD4+ T cell from ART suppressed PWH treated with pacritinib alone or in combination with PMA (50 ng/ml) + ionomycin (1 µg/ml). Mean absolute HIV-1 copy numbers (copies/ml) are represented as bar plots with individual values represented by symbols. **(B)** Calculated Bliss independence index score for the combination of pacritinib + PMAi (3 independent PWH, data from E). The negative score area under the dashed line indicates antagonism for the combined drugs. **(C)** Immune cell activation of CD4+ T cells from PWH (n = 6) treated or not with pacritinib (1µM) and PMA +ionomycin (PMAi, 50 ng/ml + 1 µg/ml). CD4+ T cells were stained with early (CD69) and late activation (HLA-DR and CD25) markers 72 h post treatment with compounds and frequency of positive CD4+ T cells were assessed by flow cytometry. Data obtained from at least three independent experiments *p<0.05; **p<0.005.

To evaluate the latency promoting capacity of pacritinib *ex vivo*, resting CD4+ T cells isolated from three PWH under ART were treated with pacritinib, either alone or in combination with PMA and ionomycin (PMAi). Viral RNA levels in the culture supernatant were measured by an ultra-sensitive nested qPCR. Participant characteristics are summarized in **Supplementary Table 1**. Consistently with *in vitro* findings, pacritinib treatment abrogated PMAi-induced HIV-1 latency reversal *ex vivo* in all three subjects tested, in a comparable manner to that observed in non-clonal *in vitro* cell line models. Moreover, when spontaneous HIV-1 reactivation was detected in unstimulated CD4+ T cells, pacritinib as a single agent effectively blocked this reactivation (**Figure 2A**). Bliss independence index score (Δf_axy_) was calculated for all replicas to quantitatively assess the interaction between pacritinib and PMAi. According to the model, a negative score indicates an antagonistic effect for the combination, further confirming the LPA activity of the JAK2 inhibitor pacritinib (**Figure 2B**). An ideal LPA should block HIV transcription without induction of undesired global T-cell activation which may result in clonal expansion of latently infected CD4+ T cells contributing to HIV disease progression (35). To evaluate whether pacritinib treatment impacts CD4+ T cell activation, primary CD4+ T cells were isolated from seven ART-suppressed PWH and treated *ex vivo* with pacritinib or PMAi as a positive control of immune activation. Markers for early and late immune activation were evaluated by flow cytometry 72h post treatment (**Supplementary Figure 1**). In contrast to PMAi, *ex vivo* treatment with pacritinib did not induce immune activation of CD4+ T cells, as measured by the frequency of the early activation marker CD69+ and the late activation markers CD25+ and HLADR +. Indeed, a significant reduction in the frequency of CD25+ CD4+ T cell population was observed when compared to the unstimulated condition (p=0.034) (**Figure 2C**).

Together, these results suggest that JAK2i pacritinib acts as an LPA, which inhibits HIV-1 reactivation in multiple types of reservoir cells without inducing immune activation.

### Pacritinib exerts its LPA activity through the downregulation of IRF7 expression

To further characterize the mechanisms underlying pacritinib’s LPA capacity, we performed whole transcriptomic profiling of the HL-HIG model treated with PMA, fedratinib and pacritinib. Our computational analysis of the differentially expressed genes (DEGs) revealed minimal genetic changes in pacritinib-treated cells compared to the untreated control (**Supplementary Figure 2A**), contrary to the LRAs-treated conditions. Previously, we have demonstrated that the JAK2i, fedratinib exerts its LRA activity through an interferon-independent activation of interferon regulatory factor 7 (IRF7) (21). We then analyzed the DEGs data with Qiagen’s IPA software to visualize or predict the activation status of hallmark genes of the response of IRF7 to RNA viruses and their implication on the innate immune response and HIV-1 replication. In the pacritinib-treated condition, most of the genes were, or were predicted to be, effectively downregulated in contrast to PMA and fedratinib conditions (**Supplementary Figure 2B**).

**Figure 3:**
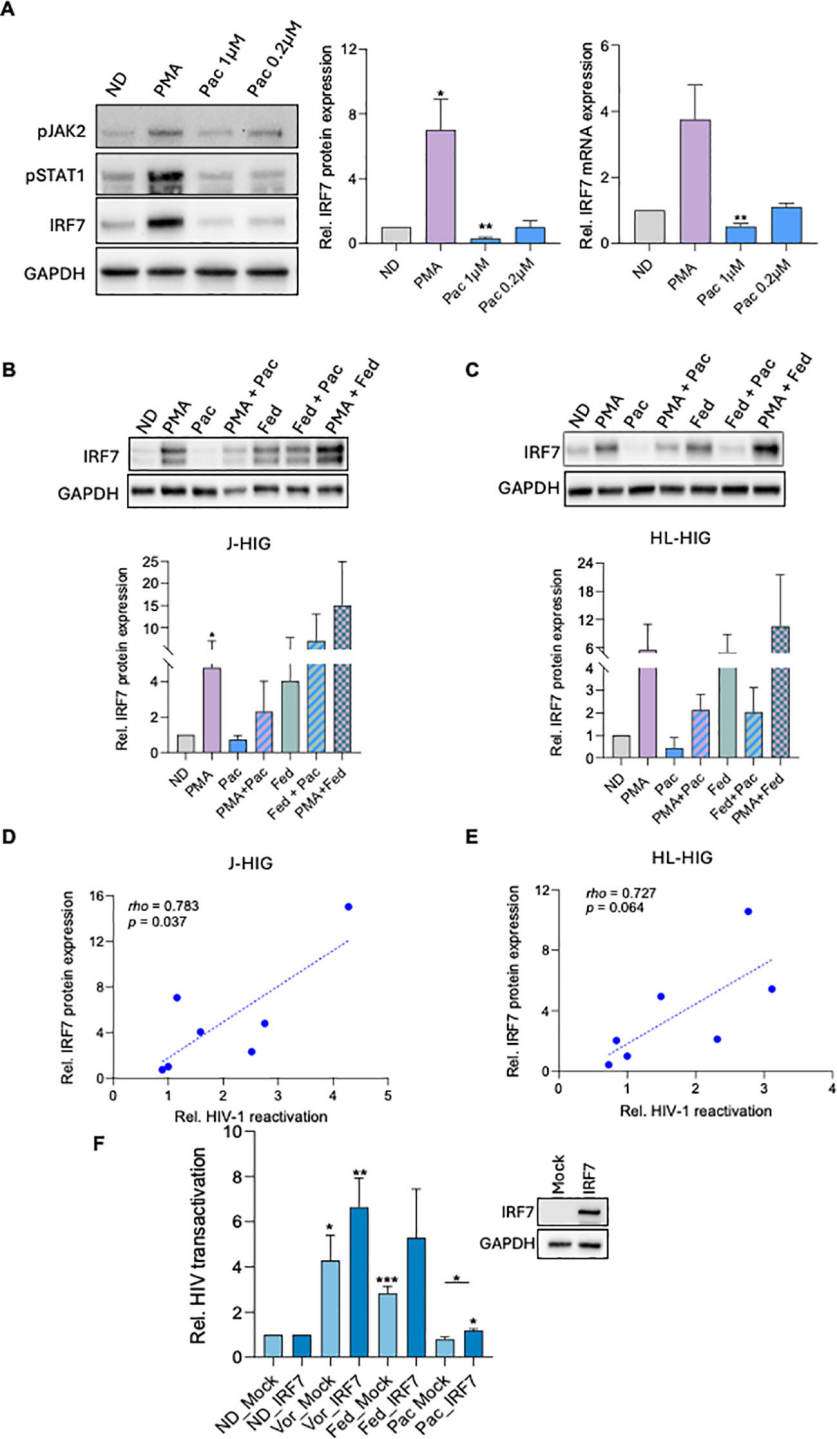
Pacritinib blocks HIV latency reversal in an IRF7-dependent manner. **(A)** Left panel, WB of a representative experiment of HL60 cells treated with PMA or with a dose response of pacritinib (Pac) immunoblotted with anti-pJAK2, anti-pSTAT1, anti-IRF7 and anti-GAPDH antibodies. Middle panel, bar plots represent the mean quantification of IRF7 bands obtained by densitometry analysis of three independent experiments. Values were normalized to that of GAPDH used as a loading control and relativized to the untreated condition (ND). Right panel, relative mRNA expression of IRF7 gene expression measured by quantitative RT-PCR and normalized to GAPDH. **(B, C)** WB of a representative experiment in J-HIG **(B)** and HL-HIG **(C)** cells treated with PMA, pacritinib or fedratinib (Fed), alone or in combination, and immunoblotted with anti-IRF7 and anti-GAPDH antibodies. Bar plots represent the mean quantification of bands obtained by densitometry analysis of 3 independent experiments. **(D, E)** Correlation plots of IRF7 and gene expression versus HIV-1 latency reversal capacity of PMA or JAK2i-treated J-HIG and HL-HIG cells. **(F)** Transactivation assay as measured by luciferase expression in TZM-bl cells transfected to overexpress IRF7 protein and 24 hours after drug treatment. Luciferase values were normalized within each transfection condition (mock or IRF7 overexpression, *left* panel), and overexpression of IRF7 confirmed by WB (*right* panel). PMA: phorbol 12-myristate 13-acetate (0,1µg/mL); Pac: pacritinib (1µM); Fed: fedratinib (5µM); Vor: vorinostat (5µM). Data expressed as means ± SD values from at least three independent experiments normalized to the no drug (ND) condition *p<0.05; **p<0.005; ***p<0.0001.

We next conducted a more detailed evaluation of the pathway to gain insights into the mechanism by which pacritinib exerts its latency-promoting activity. Examination of the JAK-STAT relevant genes expression by western blot indicated that pacritinib treatment caused a slight decrease in pJAK2 and pSTAT1 protein levels, along with a notable dose-dependent reduction in IRF7 both at protein (p=0.002) and transcript (p=0.004) levels, in HL-60 cells (**Figure 3A**).

IRF7 expression levels have been positively correlated with the HIV latency reactivation capacity induced by JAK2 inhibitors, as well as by other common LRAs (21). Treatment with the LRA fedratinib induced HIV latency reversal as well as IRF7 mRNA upregulation in HL-HIG cells in a dose-dependent manner (**Supplementary Figure 3A**). Hence, we further evaluated the relationship between HIV-1 latency reversal blockade by pacritinib and IRF7 downmodulation in our models. We observed that IRF7 upregulation occurs at both the protein and mRNA levels in cells treated with the LRAs PMA and fedratinib, whether used individually or in combination, as expected. In contrast, when the LPA pacritinib was combined with these LRAs, it counteracted the IRF7 upregulation. (**Figures 3B, C**; **Supplementary Figure 3B**). Furthermore, IRF7 modulation directly correlated with the latency-reversing capacity of the tested compounds, either alone or in combination, significantly in the J-HIG lymphoid model (rho = 0.783, p = 0.037), and with a similar trend in the myeloid HL-HIG model (rho = 0.727, p = 0.064) (**Figures 3D, E**; **Supplementary Figure 3C**). Altogether, our data consistently support a conserved IRF7-dependent mechanism underlying HIV latency in both lymphoid and myeloid cell models.

It has been previously suggested that members of the IRF family are able to activate HIV-1 LTR transcription (36). Indeed, we have previously shown that IRF7 expression levels significantly correlated with Tat-mediated transactivation (21). To further explore pacritinib’s role as a modulator of HIV LTR transactivation, we performed a transactivation assay (**Figure 3F**). TZM-bl cells, which harbor an integrated copy of HIV-1 LTR controlling luciferase reporter gene expression, were transfected with an IRF7 expression plasmid and subjected to treatment with the LRAs vorinostat and fedratinib, along with pacritinib. In non-transfected cells, treatment with vorinostat and fedratinib led to a significant increase in luciferase expression (p=0.026; p=0.0009), whereas pacritinib treatment did not produce the transactivation of the LTR. Importantly, when IRF7 was overexpressed, HIV-1 LTR transactivation was significantly enhanced upon vorinostat and also with pacritinib treatment (p=0.005; p=0.037), following a similar pattern with fedratinib. This indicates that exogenous IRF7 overexpression counteracts the pacritinib-induced downregulation of IRF7, while pacritinib itself does not impact HIV-1 LTR transactivation in TZM-bl cells, where baseline LTR activity in the absence of stimuli is extremely low. Overall, these results provide further evidence of the direct effect of IRF7 expression on HIV-1 latency modulation and suggest that the LPA pacritinib can inhibit HIV latency reversal by downregulating IRF7, likely through an LTR transcriptional blockade.

### Pacritinib selectively inhibits multiply spliced HIV-1 transcription driven by IRF7

**Figure 4:**
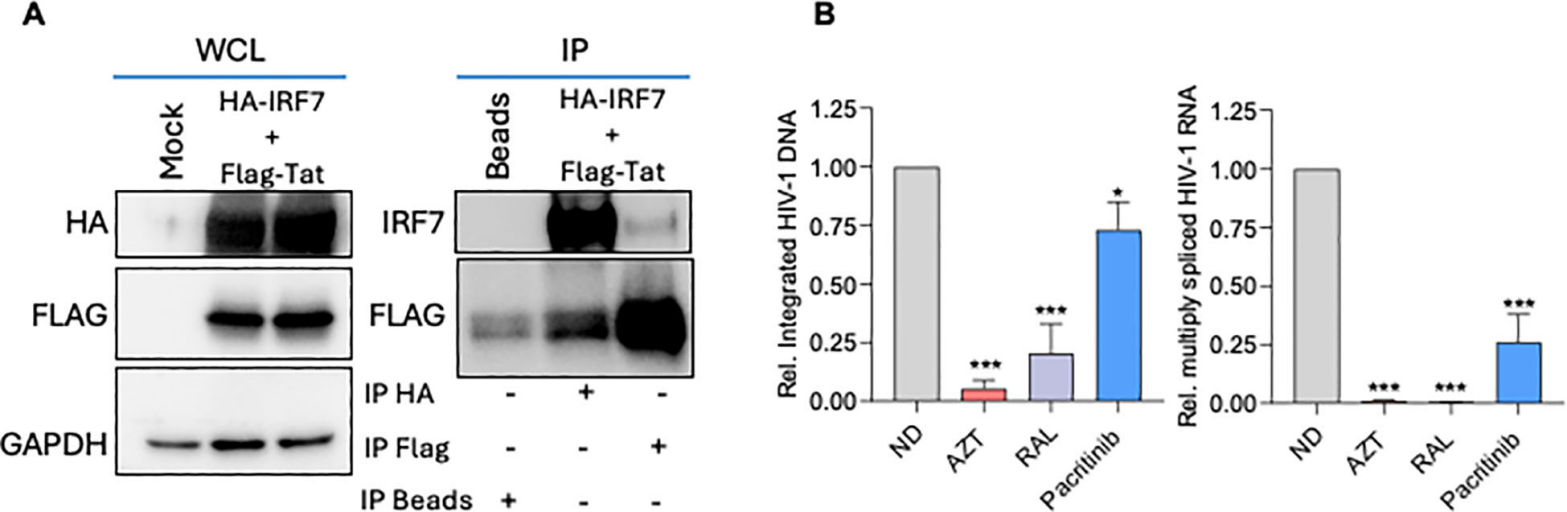
Pacritinib selectively inhibits the transcription of multiply spliced HIV-1, in which IRF7 is involved. **(A)** Immunoblots of a representative experiment showing whole cell lysates (WCL) and lysates from mock transfected cells or cells cotransfected with the two expression plasmids IRF7-HA and HIV Tat-Flag and subjected to immunoprecipitation (IP) with antibodies anti-HA, anti-Flag or beads only, blotted with anti-HA, anti IRF7, anti-Flag and anti-GAPDH antibodies. **(B)** Left panel, qPCR quantification of HIV-1 integrated DNA in CD4+ T cells from healthy donors infected with a NL4–3 HIV-1 virus treated or not with pacritinib. Right panel, qPCR quantification of HIV-1 multiply spliced transcripts of CD4+T cell from the same donors. Data represents Mean ± SD values from three independent infections in donors normalized to the no drug condition (ND). Zidovudine and raltegravir were included as control for RT and Post-RT steps. Pacritinib (1µM); AZT: zidovudine (3µM); RAL; raltegravir (2µM). *p<0.05; ***p<0.0001.

To investigate potential direct interactions between IRF7 and the viral LTR transactivator Tat, HEK293T cells were transiently co-transfected with plasmids encoding FLAG-Tat and IRF7-HA fusion proteins. Independent protein immunoprecipitations of Tat and IRF7 were performed using FLAG-specific and HA-specific agarose beads, respectively. Interestingly, IRF7 co-immunoprecipitated with FLAG-Tat and, despite the interaction being moderate, it was not observed when only beads were used as a control. Conversely, IRF7 immunoprecipitation with anti-HA beads led to the detection of co-immunoprecipitated Tat protein (**Figure 4A**), ruling out non-specific binding. Overall, these results indicate that Tat associates with IRF7 in cultured cells, providing further support for the role of IRF7 as a modulator of HIV-1 transcription. We then investigated the role of pacritinib before and during viral transcription. To this end, the levels of integrated proviral DNA and viral transcripts were quantified by qPCR. CD4+ T cells were *ex vivo* infected with an NL4–3 HIV-1 strain, and viral integration as well as viral mRNA transcription were quantified 18h and 48h post-infection, respectively. As expected, the HIV-1 reverse transcription inhibitor AZT and the HIV integration inhibitor raltegravir (ral) effectively inhibited both viral integration (AZT p< 0.0001; ral p= 0.0004) and the transcription of multiply spliced viral transcripts as measured by the amplification of HIV-1 multiply-spliced tat-rev-nef transcript (AZT, ral p< 0.000104). By contrast, pacritinib treatment had little effect on the HIV-1 integration cycle (p= 0.028), whereas a clear reduction in viral multiply spliced transcripts was observed following pacritinib treatment (p= 0.0004) (**Figure 4B**).

Taken together, these results indicate that the viral protein Tat associates with IRF7 in cultured cells. They also demonstrate the role of pacritinib in the inhibition of HIV transcription, likely through downmodulation of IRF7.

## Discussion

Understanding the cellular and molecular environment that supports HIV-1 transcription is essential for developing effective eradication strategies. In this study, we identify pacritinib, a selective JAK2 inhibitor, as a potent latency-promoting agent (LPA), in multiple non-clonal HIV-1 latency *in vitro* models, that despite exhibiting model−dependent differences in potency, consistently blocks LRA-induced HIV−1 reactivation across lymphoid and myeloid latency models, as well as in ex vivo CD4^+^ T cells from ART−suppressed individuals. Importantly, the use of non−clonal latency models enhances the physiological relevance of our findings, as these systems better capture the heterogeneity of proviral integration sites and transcriptional states characteristic of the HIV reservoir *in vivo*, which is reflected in the inherent variability in baseline levels of spontaneous reactivation. Notably, pacritinib effectively silences HIV-1 transcription without triggering immune activation, a critical requirement for “block and lock” strategies aimed at achieving a functional cure, i.e., the long-term silencing of the viral reservoir without complete eradication.

Indeed, nowadays, despite the success of antiretroviral therapy (ART) in suppressing HIV replication, it does not eliminate the latent viral reservoir, which remains a persistent source of chronic inflammation, immune dysfunction, and potential viral rebound. In recent years, several strategies—including latency-promoting agents (LPAs), latency-reversing agents (LRAs), and gene-editing technologies— have been proposed to either silence or eradicate these reservoirs, with distinct success in preclinical studies (1, 13, 37–39). More importantly, a few individuals have achieved sustained remission following stem cell transplants, widely regarded as cases of sterilizing cure, demonstrating that a full viral eradication may be biologically possible (40–43). However, translating these isolated cases into broadly applicable therapies remains a major challenge, although a scenario where pharmacological approaches such as LPAs may offer a scalable and safe path toward durable HIV remission.

Our findings show that pacritinib effect is linked to IRF7 downregulation, a transcription factor previously linked to HIV latency reversal and innate immune activation. IRF7 plays a central role in the induction of type I interferons, particularly IFN-α. Prior data from our group and others, have demonstrated that IRF7 expression correlates with the latency-reversing capacity of JAK2 inhibitors and other LRAs, and that its modulation directly impacts HIV-1 LTR transactivation (21). While both pacritinib and fedratinib are JAK2 inhibitors, they differentially modulate IRF7 expression with opposite effects on HIV−1 transcription. Specifically, fedratinib upregulates IRF7 and promotes HIV−1 reactivation, whereas pacritinib downregulates IRF7, consistent with its latency−promoting (“block”) activity. In our models, IRF7 expression positively correlates with HIV-1 transcriptional activity, and its overexpression restores LTR transactivation even in the presence of pacritinib, indicating that pacritinib LPA activity is mechanistically linked to IRF7 suppression.

IRF7 has also been identified as a genetic modifier of HIV-1 reservoirs. A genome-wide association study revealed that specific IRF7 variants are associated with reduced HIV transcriptional activity, suggesting a host genetic influence on reservoir size and behavior (44). Here, the observed interaction between IRF7 and the viral transactivator Tat further supports a direct role for IRF7 in modulating HIV-1 transcription, potentially by influencing Tat-mediated elongation or splicing of viral transcripts. Consistent with this model, pacritinib downmodulation of IRF7 expression limits the availability of IRF7 for efficient Tat-mediated transactivation, thereby impairing Tat-driven HIV-1 transcription. Additionally, polymorphisms in IRF7 have been shown to impair IFN-α production by plasmacytoid dendritic cells in response to HIV-1, potentially affecting disease progression (45). Rare loss-of-function mutations in IRF7 have also been linked to increased susceptibility to severe viral infections, including influenza and COVID-19, highlighting its critical role in antiviral defense (46, 47).

Transcriptomic and pathway analyses reinforce this mechanism, revealing minimal gene expression changes in pacritinib-treated cells and predicted downregulation of the IRF7-related HIV-1 latency reactivation pathway. These findings reinforce the concept that IRF7 is a key regulator of HIV latency and suggest that its pharmacological targeting may offer a novel route to durable transcriptional silencing. These results also align with recent reports showing that pacritinib inhibits TLR8-mediated pro-inflammatory responses to HIV-1 RNA (48), suggesting broader immunomodulatory effects.

Unlike other JAK, we show that pacritinib selectively inhibits the transcription of multiply spliced HIV-1 transcripts, which are predictive markers of productive infection (26–28) following latency reversal. Specifically, ruxolitinib reduces viral reactivation by blocking cytokine-induced STAT signaling, while filgotinib impairs HIV splicing through intron retention (21). In contrast, pacritinib exerts its effect by inhibiting multiply spliced HIV-1 tat/rev/nef transcripts associated with later transcriptional stages, likely via IRF7-dependent mechanisms, underscoring pacritinib potential as a targeted latency-promoting agent.

Taken together, our results position pacritinib as a promising candidate for functional cure strategies. By targeting IRF7, pacritinib offers a novel and selective approach to silence HIV transcription without triggering immune activation. In this sense, one limitation of the “block and lock” strategy is the persistence of replication-competent HIV genomes within the host, posing a risk of reactivation if epigenetic or transcriptional regulation is disrupted. At present, latency-promoting agents (LPAs) require continuous administration to sustain transcriptional silencing. Nevertheless, LPAs may serve as valuable adjuncts to ART by targeting both intact and defective proviruses that contribute to chronic immune activation, thereby potentially mitigating the persistent immunological activation that underlies the development of non–AIDS-related comorbidities. The concept of inducing a state of profound viral dormancy—analogous to the silencing of endogenous retroviruses—remains a compelling avenue, particularly if more readily inducible proviruses are first eliminated through latency-reversing agents (LRAs). Future studies should explore its long-term effects, potential synergy with other LPAs and LRAs. In addition, it will be important to evaluate whether pacritinib maintains latency-promoting activity in the presence of mechanistically distinct LRAs and its applicability in clinical settings.

## Data Availability

RNA-seq data generated in this study have been deposited in NCBI GEO under accession number GSE210342. Additional datasets and materials are available from the corresponding authors upon reasonable request.
