# Supplementary material for: Modulation of IRF7-driven transcription as a strategy to control HIV-1 latency
Source: Front Immunol. 2026 Mar 16;17:1735192. doi: 10.3389/fimmu.2026.1735192 (PMC13033599; doi:10.3389/fimmu.2026.1735192)

Table S1. Immunological and virological characteristics of study participants at the time of cell sample collection

ID	Age	Sex	Ethnicity	Estimated min. length of HIV infection (years)	Estimated min. length of viral suppression (years)	CD4 Nadir (cells/µl)	CD4 count (cells/µl)	Viral Load (copies/ml)	ART Regimen*
P1	38	M	Caucasian	5	5	411	842	<40	DTG/RPV
P2	47	M	Caucasian	8	6	308	1143	<40	DRV/COBI
P3	51	M	Hispanic	10	9	445	804	<40	DRV/COBI
P4	63	M	Caucasian	13	10	448	1085	<40	FTC/RPV/TDF
P5	42	M	Caucasian	4	4	151	1078	<40	DTG/ABC/3TC
P6	39	F	Caucasian	11	9	504	1056	<40	DTG/ABC/3TC
P7	45	M	Caucasian	4	4	460	865	<40	3TC/DRV

* DTG, dolutegravir; RPV, Rilvipirine; COBI, cobicistat; FTC, Emtricitabine; TDF, Tenofovir disoproxil fumarate; ABC, abacavir; 3TC, lamivudine DRV, darunavir;

Supplementary Figure 1

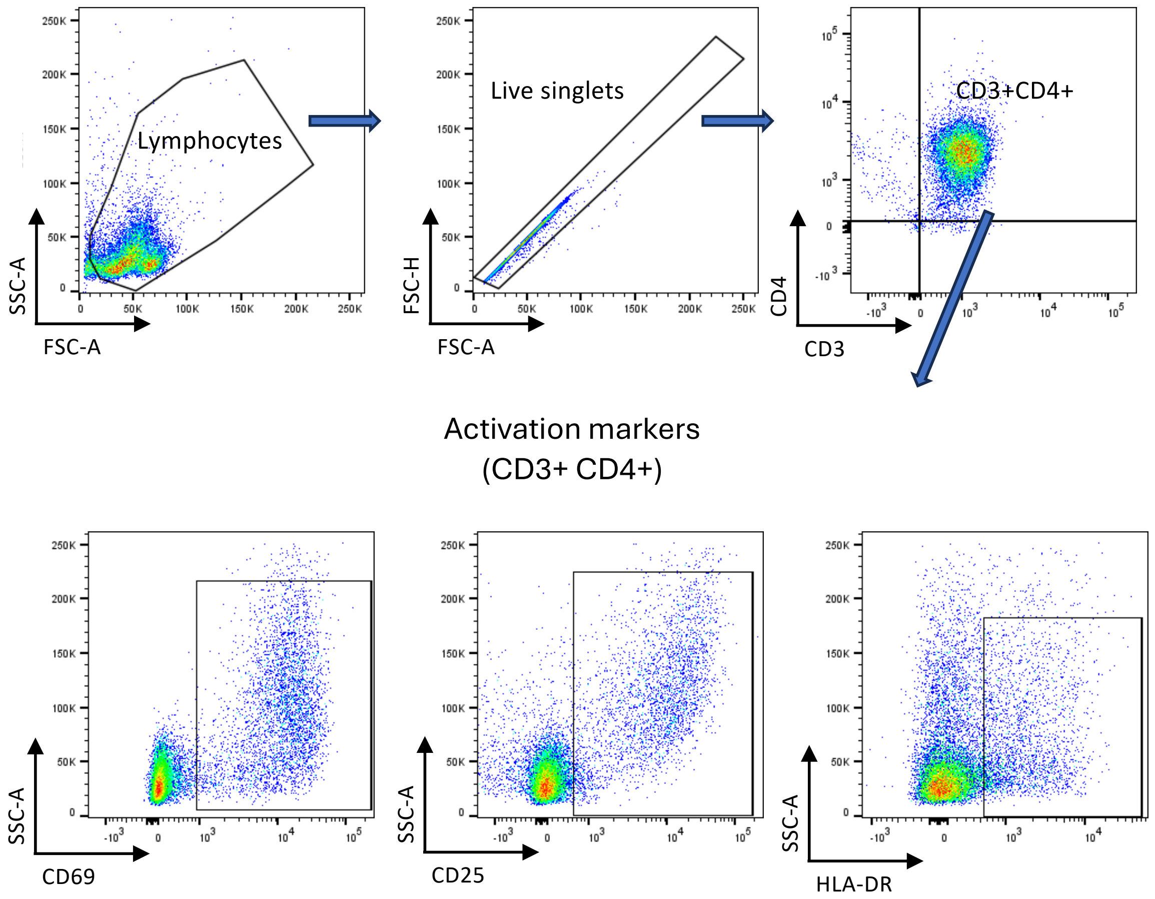

Supplementary Figure 2

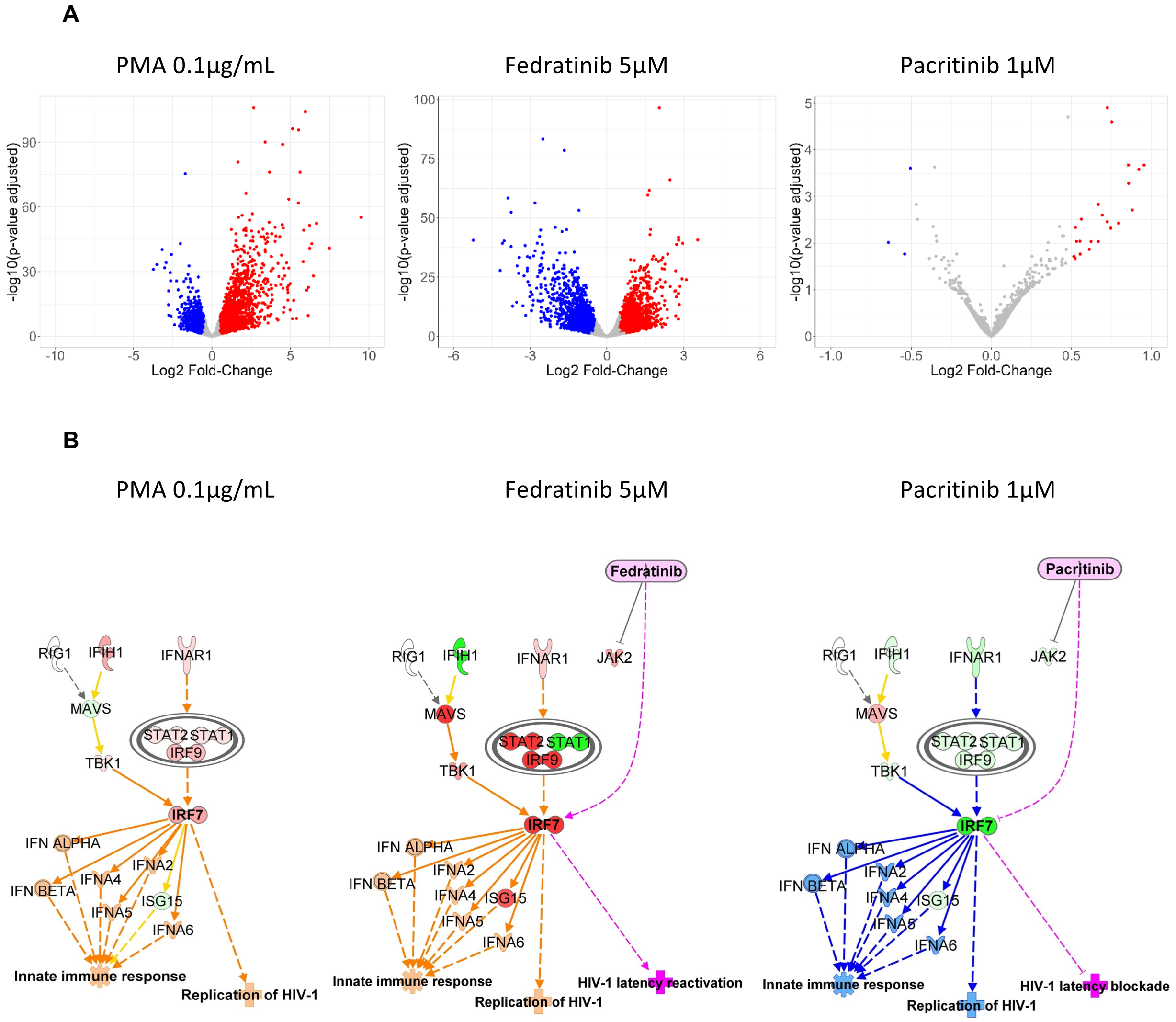

Supplementary Figure 3

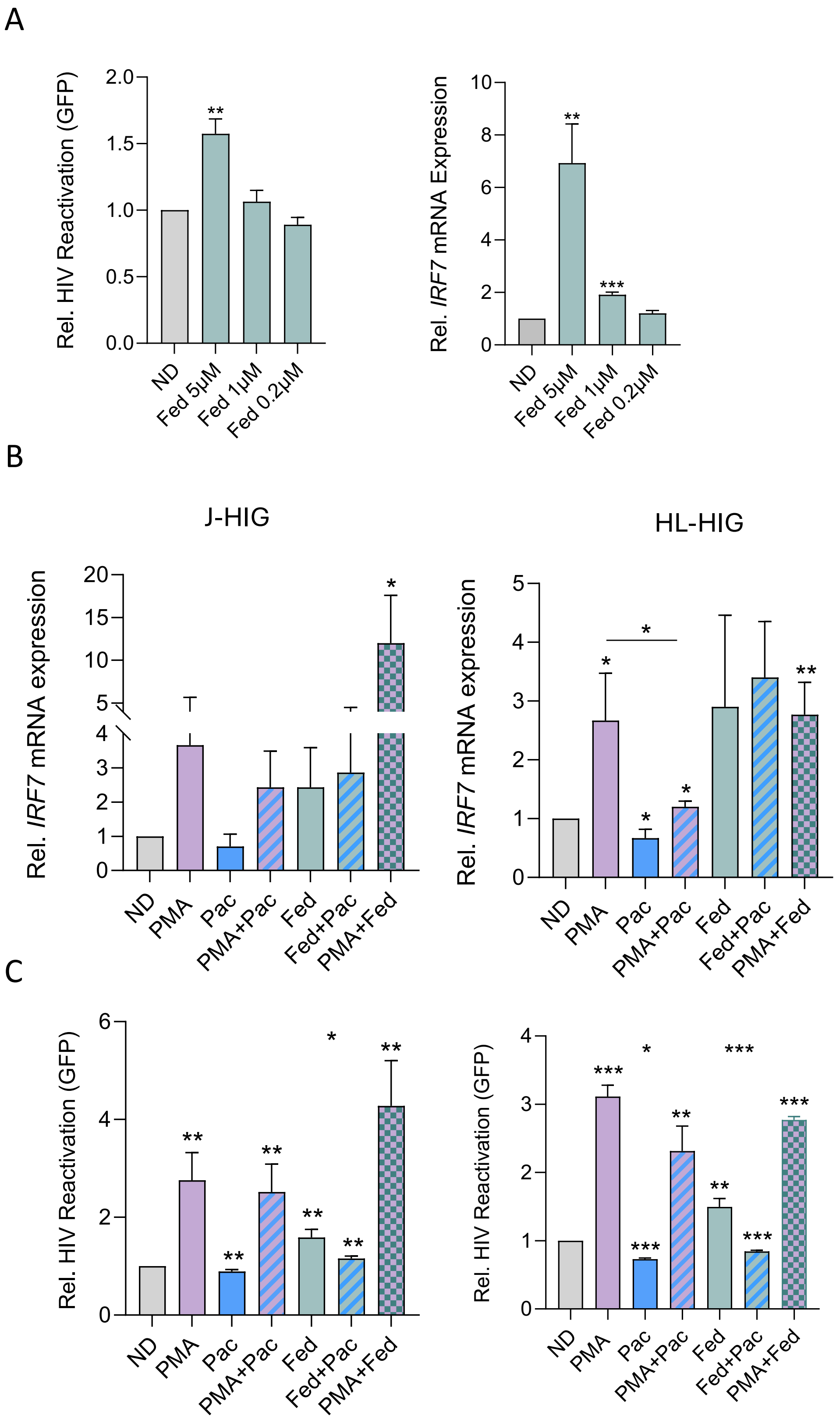

Supplementary Figure 4

A

Full length Western blot to Figure 3A

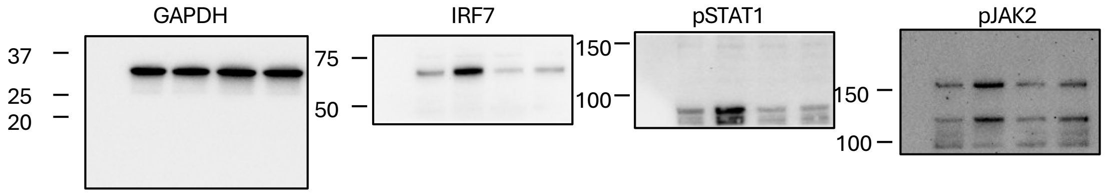

B

Full length Western blot to Fig 3B

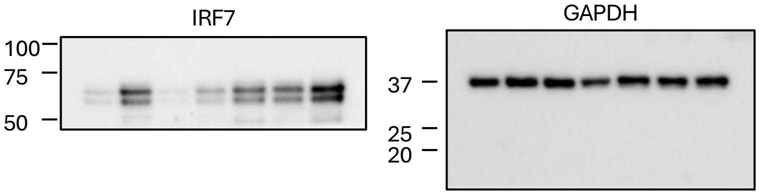

C

Full length Western blot to Fig 3C

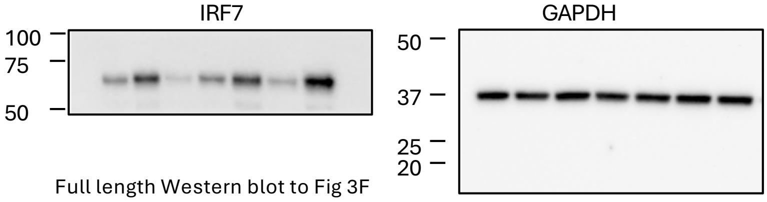

D

Full length Western blot to Fig 3F

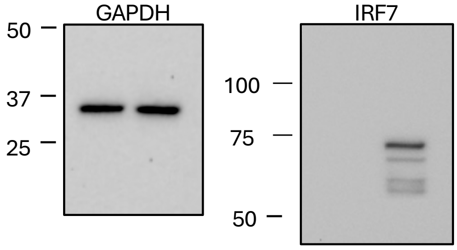

E

Full length Western blot to Fig 4A

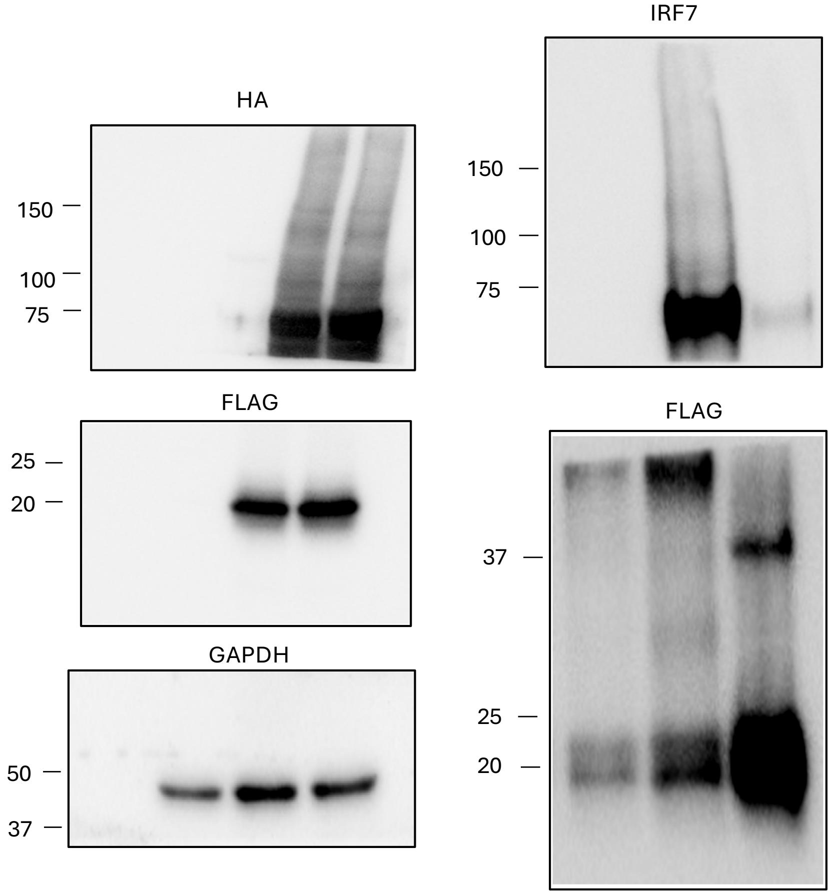

Supplement: Supplementary file 1 [file DataSheet1.pdf]
